# Cardiac Sarcoma Mimicking Libman–Sacks Endocarditis in a Patient with Systemic Lupus Erythematosus (SLE): A Case Report and Literature Review

**DOI:** 10.3390/jcm13154345

**Published:** 2024-07-25

**Authors:** Einat Ritter, Tamar Itach, Daphna Paran, Aleksandr Gaskin, Ofer Havakuk, Jacob Nadav Ablin

**Affiliations:** 1 Department of Gastroenterology and Liver Diseases, Tel Aviv Sourasky Medical Center, Tel Aviv 64239, Israel; 2 Department of Cardiology, Tel Aviv Sourasky Medical Center, Tel Aviv 64239, Israel; 3 Department of Rheumatology, Tel Aviv Sourasky Medical Center, Tel Aviv 64239, Israel; 4 Tel Aviv University Faculty of Medicine, Tel Aviv 69978, Israel; 5 Department of Internal Medicine H, Tel Aviv Sourasky Medical Center, Tel Aviv 64239, Israel; gaskin.alexandr@gmail.com

**Keywords:** systemic lupus erythematosus (SLE), Libman–Sacks endocarditis (LSE), cardiac sarcoma, clubbing

## Abstract

We present the case of a 39-year-old woman who was diagnosed with SLE and antiphospholipid antibodies 8 years ago. The chief manifestations of her disease included low-grade fever and polyarthritis. Eight months before presentation, she experienced symptoms attributed to a flare of SLE, leading to an increase in immunomodulatory treatment with no improvement. She presented to the emergency room with acute onset of dyspnea. Clubbing of her fingers and toes was noted. When questioned, she reported the onset of clubbing 5 months earlier. A CTA was performed to rule out pulmonary embolism, which was excluded, although it revealed a severely damaged mitral valve with severe insufficiency and a large mass on the valve, protruding into the left atrium. Antibiotics were started, with a working diagnosis of infectious endocarditis; however, the severe mitral valve dysfunction lead to emergency mitral valve replacement, revealing an organized thrombus. She was treated with anticoagulation, with a working diagnosis of Libman–Sacks endocarditis, with no improvement. Additional immunosuppression failed to improve her symptoms. Enlargement of the thrombotic mass and an increased gradient across the prosthetic mitral valve led to repeat surgery, culminating in a diagnosis of high-grade sarcoma within the left atrial mass. We further discuss cardiac sarcoma and describe the occurrence of clubbing in patients with sarcoma. This case highlights the importance of interdisciplinary collaboration and the need for vigilant monitoring in refractory cases, particularly when atypical presentations arise.

## 1. Case Presentation

**Figure 1:**
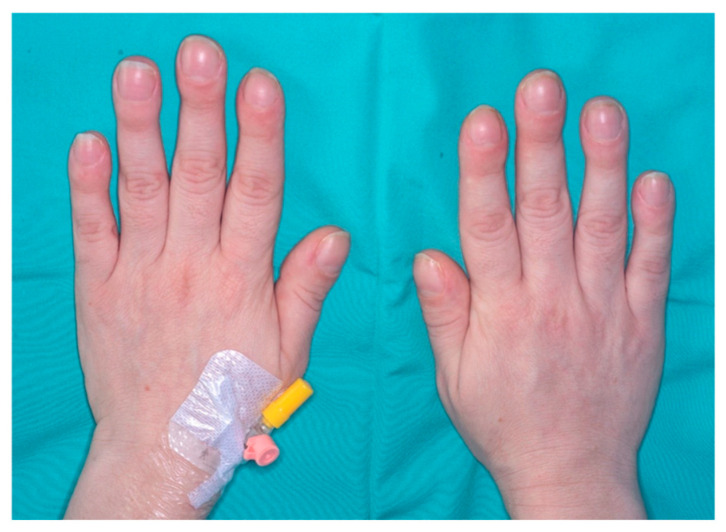
Physical examination revealed digital clubbing. Published with the permission of the rights owner.

A 39-year-old woman was diagnosed with SLE and antiphospholipid antibodies 8 years ago. The chief manifestations of her disease included low-grade fever and polyarthritis. She was treated with Plaquenil and aspirin, achieving a good clinical response for the first two years. Eight months before presentation, she experienced symptoms attributed to a flare of SLE, leading to an increase in immunomodulatory treatment with no improvement. Eight months prior to her presentation, she experienced symptoms attributed to a flare of SLE, with low-grade fever, polyarthralgia with prolonged morning stiffness, worsening anemia and markedly elevated ESR and CRP, leading to an increase in immunomodulatory treatment with no improvement. She was treated with high-dose prednisone (20 mg/day) and methotrexate (15 mg/week) with no improvement; hence, Belimumab (200 mg/week) was added to her treatment regimen. She also noted painless swelling of her fingertips and toes, and pain along her shins, over the past month (Figure 1). Two weeks prior to admission, she experienced daily fevers of 38.4 degrees, accompanied by shivers. She was referred to the emergency department due to acute-onset dyspnea to rule out pulmonary embolism (PE).

Her obstetric history was notable for two miscarriages at 12 and 13 weeks of gestation. There was no history of a thrombotic event. She had a positive lupus anticoagulant (LAC) test at the time of her diagnosis. 

Upon admission, the patient was afebrile; her blood pressure was 97/60 mm Hg, heart rate 122 beats per minute, and oxygen saturation was 100% in room air. On physical examination, her breath sounds were normal. Since she was tachycardic, it was difficult to discern murmurs on auscultation. No splinter hemorrhages or other stigmata of endocarditis were identified. Prominent clubbing was noted in the digits of the upper and lower limbs. Initial laboratory evaluation demonstrated normochromic normocytic anemia (hemoglobin 8.2 g/dL), with normal leukocyte and platelet counts, PT 12.7 s. The blood smear showed no signs of hemolysis, and both the bilirubin and Coombs tests were normal. The blood chemistry results indicated normal electrolytes, liver, and kidney function tests, with mild hypoalbuminemia (albumin 3.4 g/dL). The C-reactive protein (CRP) level was markedly elevated 150 mg/L (normal value, <5), and the D-dimer test was mildly elevated (8 μ/mL). her electrocardiography was remarkable for sinus tachycardia, with no evidence of ischemia or right ventricular strain.

**Figure 2:**
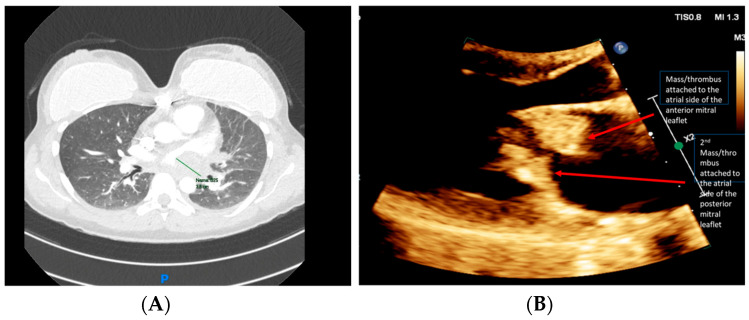
(**A**) Chest CT revealed a large filling defect extending from the left atrium to the left hilum; and (**B**) TTE (parasternal long axis) demonstrated multiple mobile masses attached to the mitral valve. Published with the permission of the rights owner.

A chest computed tomography angiography (CTA) was performed, which did not demonstrate evidence of PE. The imaging revealed a large left atrial (LA) filling defect, with signs of pulmonary hypertension (Figure 2A). Transthoracic echocardiogram (TTE) demonstrated multiple mobile mitral valve masses (anterior and posterior), mostly on the atrial side, 29 × 16 mm in size, prolapsing into the left ventricle (LV) and causing severe functional mitral stenosis (MS), mitral regurgitation (MR), and pulmonary hypertension (Figure 2B). 

**Figure 3:**
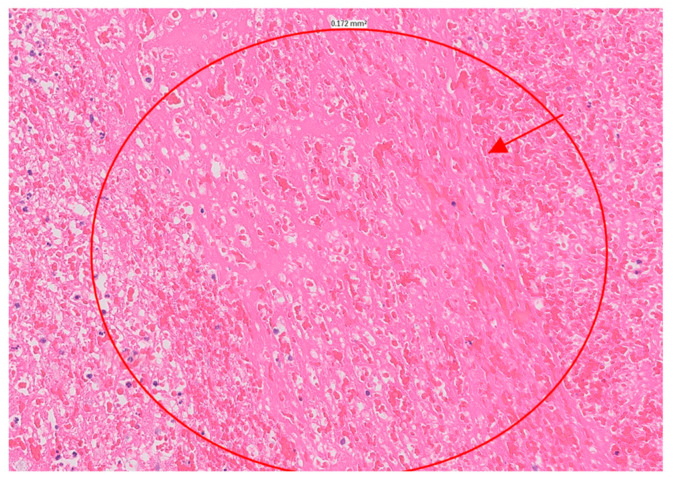
Histology demonstrated a large organizing thrombus, fibrin and acute inflammation.

At this point in the patient’s course, the likelihood of infectious endocarditis was considered significant. Blood cultures and serologic markers were drawn, and broad-spectrum antibiotics were initiated. Due to the severity of the MR, which was accompanied by acute heart failure, the patient was referred for urgent surgical intervention and underwent mitral valve replacement within 24 h of her admission. During surgery, two large masses of tissue attached to the posterior annulus of the mitral valve were noticed. The masses appeared to be blocking the valve opening and infiltrating the posterior leaflet, posterior annulus, and surrounding cardiac tissue. The cardiac surgeon noted that the left atrial wall was infiltrated, suggesting a tumor. However, the pathologic evaluation revealed a large, organized thrombus, with no evidence of malignancy (Figure 3). Blood cultures, as well as serological tests for Coxiella burnetii and Bartonella henselae, were negative, as was a pan-bacterial PCR of the surgical sample. Serological tests for antiphospholipid autoantibodies (aPL) were negative, including anticardiolipin antibodies, anti-β2 glycoprotein-1 antibodies as well as a LAC test. Antinuclear antibodies were positive at a titer of 1/640, with a homogeneous and speckled pattern. Anti-dsDNA antibodies were positive at a titer of 1/160, while C3 and C4 were within the normal range.

The patient was diagnosed with marantic (Libman–Sacks endocarditis (LSE)) endocarditis. LSE is a form of nonbacterial thrombotic endocarditis often associated with SLE. It is characterized by the presence of sterile vegetations on the heart valves, which can lead to embolic events and other complications. Given this diagnosis, antibiotics were discontinued [1,2]. Treatment was started with anticoagulation (low-molecular weight heparin, 60 mg/BID and subsequently warfarin 5 mg/day). Concurrently, an anti-inflammatory regimen was initiated, with a course of oral corticosteroids (Prednisone 15 mg). Rapid post-surgical clinical and laboratory improvement was observed, with resolution of fever, improvement of arthralgia, normalization of CRP, and improvement of hemoglobin levels. The clubbing of her fingers and toes remained unchanged. A TTE performed two weeks later revealed the normal function of the prosthetic mitral valve, with no evidence of vegetation. 

Three months later, the patient was again referred to the emergency department due to severe pain in her left shoulder and arm. Upon admission, she was tachycardic, tachypneic and hemodynamically stable. Laboratory assessment showed an elevated CRP and D-Dimer test, while the INR was within the therapeutic range (INR-3). Table 1 summarizes the laboratory test results from both hospital visits. Repeated CTA revealed large filling defects, approximately 36 mm wide, extending from the LA to the left hilum, surrounding vessels, and bronchi, and partially compressing them, without evidence of complete occlusion. TTE demonstrated severe LA dilatation, with a large mass in the LA and a normal pressure gradient across the prosthetic mitral valve. 

A multidisciplinary team meeting was conducted, including specialists in the fields of cardiology, thoracic surgery, rheumatology, infectious disease, and pulmonary medicine. The possibility of obtaining a histological specimen was discussed, either through an endobronchial ultrasound (EBUS) with trans-bronchial needle aspiration, through an endovascular approach, or by performing a repeated surgical intervention. After carefully reviewing the imaging findings and weighing the possibilities, all such approaches were deemed to carry a prohibitive risk for the patient, with uncertainty regarding their diagnostic yield. Thus, as the leading working diagnosis continued to focus on an inflammatory–thrombotic process within the LA, with insufficient control under the hitherto administered anti-inflammatory and anticoagulant treatment, a course of intensified anti-inflammatory treatment was chosen. The patient was subsequently started on a regimen of pulse Methylprednisolone (1000 mg/d for 3 days), Rituximab (1000 mg, 2 dose, 2 weeks apart) and enoxaparin (80 mg ∗ 2 day). 

Follow-up echocardiography one month later revealed a substantial left atrial mass, protruding from the pulmonary vein (measuring 9.5 cm^2^), and a moderately elevated gradient across the mitral valve prosthesis (16/10 mmHg). A revision of the pathologic specimen to rule out a malignant process revealed a thrombus, with no evidence of malignancy. A repeat chest CT showed findings similar to those previously described. In view of the poorly controlled, life-threatening process, treatment with IV cyclophosphamide was considered as a salvage therapy but was not initiated [3].

**Figure 4:**
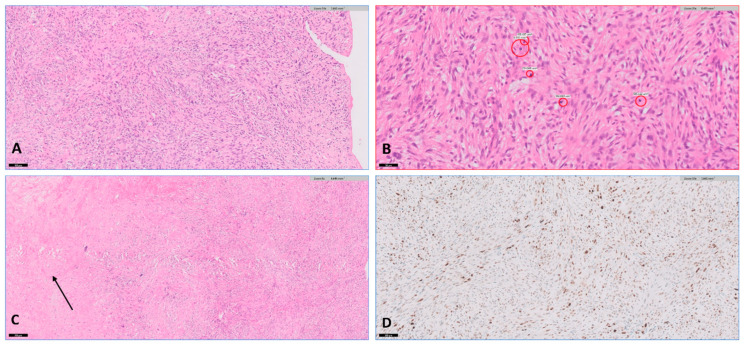
Histology of the excisional biopsy, fragments of a mesenchymal lesion composed of epithelioid and spindle cells showing moderate to severe atypia (**A**) with brisk mitotic activity (**B**), including atypical figures and areas of necrosis (arrow) (**C**). Immunohistochemistry reveals the presence of diffuse MDM2 positivity (**D**).

A repeat echocardiogram after a month revealed a very large, multilobar, redundant mass occupying most of the LA cavity, with a severely elevated pressure gradient across the valve. Considering the absence of a response to treatment and the evident clinical deterioration, the decision was made to proceed with an exploratory surgical procedure. During surgery, a large gelatinous, mostly encapsulated mass was observed in the LA, which was excised down to its base. As the mechanical valve appeared intact and did not seem to be the source of the process, it was decided not to replace it. Specimens were sent for pathological and microbiological examination. The pathology report revealed epithelioid and spindle cells with moderate to severe atypia and areas of necrosis, and diffuse Murine double minute 2 (MDM2) positivity on immunohistochemistry, consistent with high-grade sarcoma (Figure 4). Cardiac sarcoma is a rare and aggressive malignant tumor of the heart. Due to its rarity and nonspecific symptoms, it is often difficult to diagnose. At the time of writing, the patient has completed a combination of chemotherapy (Adriamycin and Ifosfamide) and radiotherapy (28 fractions, totaling 56 Gray). She has a good response, and the digital clubbing has partially regressed.

## 2. Commentary and Discussion

Wiliam Osler is famously quoted as saying “The good physician treats the disease; the great physician treats the patient who has the disease”. While this epitaph is generally construed as pointing to the necessity of maintaining a holistic and humanistic approach toward our patients, it also poignantly reminds us to remain vigilant when patients classified with a particular diagnostic label appear to be evolving into something different. Treating patients with SLE is notoriously challenging, due to both the broad clinical spectrum of this multisystem autoimmune disorder and the unpredictable clinical course. Some patients might initially exhibit severe, and potentially fatal, symptoms like catastrophic antiphospholipid syndrome (CAPS) or diffuse alveolar hemorrhage [4,5]. Others, however, may experience a milder chronic disease course, characterized by joint pain and fatigue. In these cases, clinical care aims to attain remission or, if unachievable, maintain low disease activity. Nonetheless, even relatively mild cases may develop exacerbations, as well as novel clinical manifestations, requiring constant vigilance on the part of their physicians. In the current case, a patient suffering from relatively mild, chronic SLE presented with worsening fatigue, low-grade fever and arthralgia, accompanied by elevated inflammatory markers, despite receiving escalating regimens of SLE treatment. 

SLE may present with diverse cardiac manifestations, including atherosclerosis, thrombosis, arrhythmias, pericarditis and more [6]. While several pulmonary complications of SLE might explain the shortness of breath, progressive clubbing is not commonly observed in SLE and was a clue to the fact that something different may be developing [7]. In the case described, the patient’s previous diagnosis of SLE and positive LAC antibodies several years prior to her presentation appeared to point in specific diagnostic and therapeutic directions, which ultimately proved inaccurate. The finding of a large thrombotic process within the cardiac chambers was interpreted as representing LSE, an inflammatory–thrombotic process, and was thus treated with anticoagulation and escalating anti-inflammatory medications. This appeared reasonable, since both the imaging and the surgical specimen failed to demonstrate the malignancy eventually uncovered. The patient’s history of previously positive LAC antibodies, now showing negative serology, was attributed to either an initial false laboratory result or, more plausibly, the excessive consumption of antibodies during an acute thrombotic event, as documented in the literature [8]. However, at least in retrospect, the unusual manifestation of marked clubbing may have been the non-fitting clue that indicated a divergent diagnosis. Primary cardiac tumors (PCTs) are rare, with an approximate incidence of 0.02%. The majority (85–90%) of them are benign. Of the malignant tumors, sarcomas are the most common (65%), followed by lymphoma and mesothelioma (27% and 8%, respectively) [8]. There has been a notable increase in the incidence of PCT over the past decade, mainly attributed to advances in imaging techniques [9]. As cardiac tumors can be asymptomatic or present symptoms that resemble other conditions, it poses a clinical challenge both to entertain the possibility of such a tumor and to conduct appropriate diagnostic testing. In general, the signs and symptoms of PCT are determined by the tumor’s location rather than its histology and may arise from embolism, obstruction, valvular dysfunction, or direct myocardial invasion [10]. Radiologic hints of malignancy include local tumor invasion, hemorrhage or necrosis, rapid tumor growth, involvement of more than one cardiac chamber, rapid growth, and pericardial effusion. 

The median age at diagnosis of cardiac sarcoma is 45 years [11]. All the histopathologic types of sarcoma have been described, while angiosarcoma is the most common (43%) [12]. The most common sites involved are the right and left atria, right ventricle, and LV (in decreasing order of frequency) [13]. Similar to the majority of primary malignant cardiac tumors, cardiac sarcoma carries a poor prognosis, with a median survival of 9 months [14].

The patient described here presented with digital clubbing, which preceded the onset of the other symptoms by several months. Digital clubbing, identified on physical examination by the loss of the normal angle between the nail plate and the skin, is a manifestation of hypertrophic osteoarthropathy, caused by proliferation and edema of the connective tissue. The most common form of clubbing is acquired, painless and bilateral. Clubbing was first described in association with emphysema by Hippocrates, who wrote: “The nails of the hand are bent; the fingers are hot, especially in their extremities”, and indeed, the literature review indicated that up to 80% of cases are associated with cardiovascular or chronic pulmonary disease, followed by chronic gastrointestinal or rheumatologic disease [15]. The mechanism for the development of clubbing remains unclear, and several hypotheses have been suggested to explain it. The theories include the contribution of genetic factors, the effects of hypoxia, as discussed further, higher levels of plasma growth factors in patients with clubbing, and the idea that clubbed digits are the embryonic claws we have lost [16]. Probably the most promising theory was proposed by Martin and Dickinson in 1987 [17]. According to their hypothesis, the platelet clusters and macrophages found in the distal digits release factors such as platelet-derived growth factor (PDGF), leading to increased permeability and connective tissue changes, eventually resulting in clubbing formation. Similarly, Atkinson et al. described the role of vascular endothelial growth factor (VEGF) in the pathophysiology of clubbing [18]. Hypoxemia, as well as the tumor mass itself, likely plays a significant role in releasing these factors [19].

While clubbing is a known paraneoplastic sign, it has rarely been described in association with cardiac sarcoma. Ung et al. have recently described a 52-year-old female patient with acute onset of clubbing, inflammatory polyarthralgia and a new heart murmur, who was diagnosed with LA intimal sarcoma with positive MDM2 staining. The clubbing in this case improved rapidly after the tumor resection and commencement of chemotherapy [20]. Another case report describes a 52-year-old non-smoking male evaluated for progressive dyspnea and exercise intolerance, accompanied by digital clubbing over the prior 2–3 months. The patient had a history of chronic pulmonary hypertension due to thromboembolic disease, which is usually not associated with clubbing. Similarly to our case, this could be an alarming sign and raise the possibility of a different diagnosis. During the evaluations, the patient was diagnosed with pulmonary artery spindle cell sarcoma [21]. Table 2 summarizes the three cases of digital clubbing and sarcoma. 

Another point to consider is the association between SLE and sarcoma. Studies show that patients with SLE have a higher overall risk of developing cancer. For instance, a registry from Finland’s National Health Insurance reports a hazard ratio of 1.4 (95% CI: 1.1–1.8) for any malignancy compared to the general population [22]. A systematic review by Clarke et al. analyzed 41 studies and found a relative risk of 1.18 (95% CI: 1.00–1.38) for malignancy in SLE patients. The highest risks were observed for Hodgkin’s lymphoma, myeloma, and cancers of the cervix, bladder, and thyroid, while the other malignancies had a lower risk [23]. Several case reports of Kaposi sarcoma in SLE have been described and may be related to immunosuppressive drugs [24,25]. To the best of our knowledge, we describe here the first case of cardiac sarcoma in a patient with SLE. 

LSE is a rare and incompletely understood manifestation of SLE. While it is intuitive to think of the thrombotic complications of SLE, especially in the context of documented hypercoagulability such as APS, it is less clear why such pathology may develop due to the lack of evidence of such a pro-thrombotic predisposition and to what extent a local inflammatory process is involved [26,27]. Moreover, the optimal treatment of LSE beyond anticoagulation remains unclear. When SLE causes life- or organ-threatening complications, it is treated with immunosuppressive medications such as cyclophosphamide and rituximab, but the progressive intracardiac process observed could not be classified among the major complications of SLE. Clinical flexibility and close follow-up are imperative in such perplexing cases and did eventually lead to a precise diagnosis and treatment.

## 3. Conclusions

Systemic lupus erythematosus (SLE) is a disease with diverse manifestations and an unpredictable course. In this article, we describe a unique case where progressive clubbing, accompanied by dyspnea and low-grade fever, ultimately led to a diagnosis of cardiac sarcoma. This case highlights the importance of considering rare diseases, including malignancies, in the differential diagnosis. Additionally, it emphasizes the critical role of a multidisciplinary team working on decision-making for complex cases.

## Figures and Tables

**Table 1:** Summary of the initial laboratory results.

Parameter	First Episode	3 Months Later
Hb (g/dL)	**10.7**	**11.7**
WBC (×10 × 10^3^/uL)	9.1	6.6
PLT (×10 × 10^3^/uL)	168	221
INR	1.19	3.02
PTT	30.2	55
Cr (mg/dL)	0.55	0.53
Tot. protein (g/L)	**46.8**	72.4
Albumin (g/L)	**27**	42.6
Urine protein	NEG	NEG
ANA	>1:160	>1:160
dsDNA (U/mL)	**43.2**	-
C3 (g/L)	1.53	1.59
C4 (g/L)	0.340	0.360
CRP (mg/L)	**97**	**70.4**
ALT (U/L)	13	14
AST	-	12
LDH (U/L)	**684**	**479**

Hb—hemoglobin; WBC—white blood cell; PLT platelet count; PTT—partial thromboplastin time; Cr—creatinine; DsDNA—anti-double-stranded DNA antibodies; CRP—C-reactive protein; ALT—alanine transaminase; AST—aspartate transferase; LDH—lactate dehydrogenase.

**Table 2:** Characteristics of patients with sarcoma and digital clubbing.

Gender	Age (Years)	Past Medical History	Tumor Location	Onset of Clubbing Preceding the Diagnosis	Resolution of Clubbing after Treatment
Female ^1^	52	-	LA intimal (spindle cell) sarcoma	3 months	Complete resolution 2 weeks after tumor resection
Male ^2^	53	Pulmonary hypertension	Pulmonary artery spindle cell sarcoma	2–3 months	Unknown
Female ^3^	39	SLE	LA intimal (spindle cell) sarcoma	1 month	Improving

^1^: Ung et al., 2023 [20]: ^2^: Loredo et al., 1996 [21]. ^3^: Current manuscript. SLE—systemic lupus erythematosus.
